# Low-Cycle Fatigue Behavior and the Combined Cyclic Hardening Material Model of Plate-Shaped Zn-22Al Alloy for Seismic Dampers

**DOI:** 10.3390/ma17092141

**Published:** 2024-05-03

**Authors:** Zongcheng Liu, Jianping Han, Penghui Yang

**Affiliations:** 1 School of Civil Engineering, Lanzhou University of Technology, Lanzhou 730050, China; zongchengliu@163.com; 2 School of Materials Science and Engineering, Lanzhou University of Technology, Lanzhou 730050, China; yangph@lut.edu.cn

**Keywords:** Zn-22Al alloy, low-cycle fatigue, combined cyclic hardening material, energy dissipation material

## Abstract

This study investigates the potential of the plate-shaped Zn-22 wt.% Al (Zn-22Al) alloy as an innovative energy dissipation material for seismic damping devices, since plate-shaped material is more suitable to fabricate large-scale devices for building structures. The research begins with the synthesis of Zn-22Al alloy, given its unavailability in the commercial market. Monotonic tensile tests and low-cycle fatigue tests are performed to analyze material properties and fatigue performance of plate-shaped specimens. Monotonic tensile curves and cyclic stress–strain curves, along with SEM micrographs for microstructure and fracture surface analysis, are acquired. The combined cyclic hardening material model is calibrated to facilitate finite element analysis. Experimental results reveal exceptional ductility in Zn-22Al alloy, achieving a fracture strain of 200.37% (1.11 fracture strain). Fatigue life ranges from 1126 to 189 cycles with increasing strain amplitude (±0.8% to ±2.5%), surpassing mild steel by at least 6 times. The cyclic strain–life relationships align well with the Basquin–Coffin–Manson relationship. The combined kinematic/isotropic hardening model in ABAQUS accurately predicts the hysteretic behavior of the material, showcasing the promising potential of Zn-22Al alloy for seismic damping applications.

## 1. Introduction

Metallic materials play a crucial role in mitigating seismic actions on building structures. Materials such as steel and lead are used in seismic mitigation devices like U-shaped dampers [1,2], added damping and stiffness (ADAS) dampers [3,4], buckling-restrained braces (BRB) [5,6], and lead-core rubber bearings [7].

Known for its cost-effectiveness and ease of processing, steel is commonly used in seismic mitigation. However, its relatively high yield strength, work hardening, and limited plastic deformation capability constrain its energy dissipation capabilities, leading to damage to connections and hindering the effective protection of building structures. The development of low-yield-strength steel, exemplified by materials such as LYP100 and LYP160 with respective yield strengths of 100 MPa and 160 MPa, has enhanced plastic deformation and hysteresis energy dissipation capabilities while reducing the yield strength. Xu et al., Zirakian and Zhang pointed out that during uniaxial tensile tests, these materials exhibit strains exceeding 60%, compared to the approximately 35% strain observed in common steel [8,9]. Nevertheless, Toshiaki et al. and Koichi et al. revealed that dampers made from low-yield-strength steel still experienced work hardening and strain deterioration during plastic deformation [10,11]. The yield stress of such steels can escalate under repeated loads or seismic events, requiring post-earthquake maintenance to restore their designed performance. Tanaka et al. reported that in some cases, the yield stress of dampers increased from 125 MPa to 180 MPa following a major earthquake [12]. Hence, post-earthquake maintenance is essential for these dampers to restore their original performance as designed.

Lead has been studied as an energy-dissipation material in dampers due to its low yield displacement and excellent energy dissipation capability under various dynamic loading conditions. Its ability to undergo recrystallization at room temperature indicates a capacity to revert to its initial mechanical properties after deformation, showcasing low susceptibility to fatigue [13]. However, lead toxicity, which interferes with enzyme functioning and affects various organs and systems in the human body, demands caution and restrictions in lead mining, smelting, and manufacturing of lead-containing products [14,15]. Therefore, there is a need to introduce new energy-dissipation materials with properties such as low yield strength, high ductility, stable energy dissipation capability, non-toxicity, and cost-effectiveness.

Zn-Al alloys find extensive applications in the military industry and various types of bearings. Notably, Zn-Al alloys, including Zn-22 wt.% Al (Zn-22Al), are characterized by sound processing properties, non-toxicity, environmental friendliness, and cost-effectiveness, with raw material costing only about 1/3 of copper. Zn-22Al, in particular, distinguishes itself with superior elongation, the lowest yield strength, and optimal damping performance among Zn-Al alloys, according to Zhang’s research work [16]. Research indicated that Zn-22Al alloy could achieve elongation exceeding 80% at room temperature. The production of Zn-22Al alloy involves relatively straightforward steps, such as melting, casting, heat treatment, and hot rolling. Importantly, existing factories can readily commence production without the need for additional equipment purchases.

Makii et al. suggested that, in contrast to mild steels, Zn-22Al alloy exhibited limited hardening effects under cyclic loading, resulting in stable energy dissipation capabilities [17]. Materials science research suggested that refining the grain size of Zn-22Al alloy shifted the optimal superplastic deformation conditions to high strain rates and lower temperatures, achieving superplasticity and room temperature superplasticity [18,19]. Refining the grain size is commonly achieved through techniques such as equal channel-angular extrusion/pressing (ECAE/P), thermomechanical controlling process (TMCP), and the incorporation of modifiers [20,21].

While most research and experiments on Zn-22Al alloy have focused on conclusions drawn from cylindrical specimens [22,23], seismic dampers typically use energy dissipation materials in plate form for large-scale device fabrication. In this context, TMCP is deemed more suitable for refining the grain size of Zn-22Al alloy, given its capability to produce materials in plate form.

The low-cycle fatigue (LCF) and extremely low-cycle fatigue (ELCF) failures of metallic materials in seismic dampers were critical factors leading to structural damage under seismic actions, as suggested by Fang et al. [24]. Severe structural damage occurs, especially during intense seismic actions or prolonged durations. The causes of LCF failures are attributed to complex microscopic mechanisms, including internal micro defects, lattice dislocations, fracture, and crack propagation within the metal. Due to the limited LCF resistance of common metals under large-amplitude cyclic loading, inspection, and treatment of dampers made of common metals may be required post-earthquake, and replacement might be necessary. Immediate aftershocks after the main shock make it impractical to inspect and replace seismic dampers promptly. Therefore, using energy-dissipation materials with a high fatigue life is crucial to reduce damper damage, minimize structural damage, and decrease the interruption time in structural functionality after seismic events. Makii et al.’s research indicated that Zn-22Al alloy possessed a significantly longer low-cycle fatigue life than common steel, showing potential for replacing current passive energy dissipation materials, especially in addressing LCF concerns [17].

Despite the sound energy dissipation capability and LCF life of Zn-22Al alloy, its use in structural seismic mitigation remains limited. This limitation is mainly attributed to differing research focuses between structural engineering and materials science, the non-standardized nature of Zn-22Al alloy, and its commercial inaccessibility. Limited engineering case studies, such as the use of Zn-22Al alloy seismic dampers at the Hotel Kintetsu Universal City in 2001 [25], highlighted its potential. These dampers, made of Zn-22Al alloy plates measuring 300 mm by 300 mm and with a thickness of 10 mm, inserted between steel plates and enclosed by a steel frame, were developed by Kobe Steel, Ltd. and Takenaka Corporation. The Zn-22Al alloy used is termed the world’s first successfully developed high-speed superplastic alloy, with grain size refinement to 30 nm and an elongation exceeding 100%, offering consistent performance regardless of temperature compared to oil damper counterparts [17,26].

However, important issues regarding Zn-22Al alloy still need attention. Some studies feature small test strains (e.g., ±1%), experiments conducted under high-temperature conditions (e.g., 423 K), and reliance on conclusions drawn from cylindrical specimens. A comprehensive model describing the material’s hysteresis behavior and parameters for finite element method (FEM) simulation of Zn-22Al alloy are still lacking. Therefore, further research is imperative, with a focus on structural engineering requirements, to lay the groundwork for the broader application of Zn-22Al alloy in seismic damping devices.

This study aims to address the knowledge gap and promote the application of Zn-22Al alloy as an energy-consuming material in seismic dampers by conducting a series of material-level tests to determine its basic material properties, cyclic fatigue performance, and finite element modeling parameters. The research commenced with the preparation of Zn-22Al alloy, followed by testing a total of 13 specimens, with 6 sets of data presented as the basis for subsequent analysis. These tests covered various loading modes and strain amplitudes ranging from ±0.8% to ±2.5%. The microstructure and fracture surface were investigated through SEM micrographs. The combined cyclic hardening material model was employed to characterize the cyclic behavior of Zn-22Al alloy. The model was calibrated and validated based on test results and finite element simulations.

## 2. Preparation of the Zn-22Al Alloy

**Figure 1:**
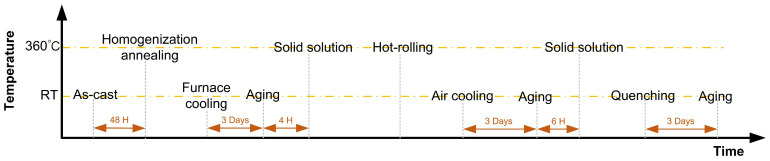
The flow chart of the preparation of the Zn-22Al alloy.

The Zn-22Al alloy used in this study was synthesized in the materials laboratory following research by Sun and Zhang [16,19], because it is not available in the commercial market. The preparation of the Zn-22Al alloy involves three main stages: casting, hot rolling, and heat treatment, as illustrated in Figure 1.

### 2.1. Casting of the Zn-22Al Alloy Ingots

The process of creating Zn-22Al alloy ingots for hot rolling involves specific steps for optimal results. Corundum crucibles are utilized, and guaranteed reagent (GR) grade aluminum and zinc particles with a diameter of 3 mm are used. Before smelting, the crucible is preheated to 200 °C in a box-type resistance furnace to remove moisture. Following a 20 min preheating period, zinc and aluminum particles are added to the crucible in a mass ratio of 78:22, and the temperature is set to 680 °C for 30 min.

After the complete melting of the metallic particles, zinc alloy slag remover and refining agent (hexachloroethane) are added separately, and the molten metal is thoroughly stirred. The slag remover reduces adhesive forces, aiding the efficient separation of metal and slag. Hexachloroethane serves as a refining agent, a common choice for degassing and refining aluminum alloys. Removal of rising slag is done using an iron spoon.

**Figure 2:**
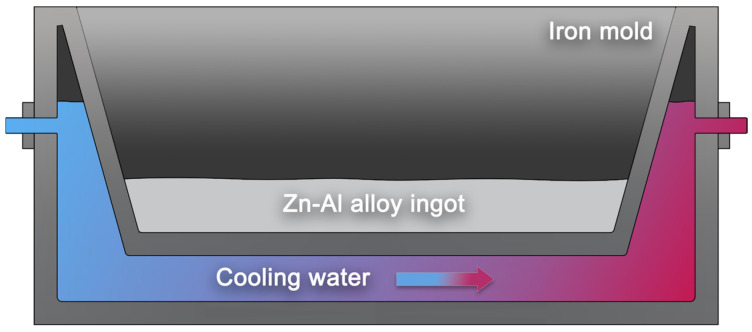
Cross-sectional diagram of water-cooled iron mold.

The corundum crucible is then returned to the resistance furnace for an additional 20 min at 680 °C, ensuring comprehensive mixing of the molten metal. In the final step, cooling water is continuously injected into the originally designed water-cooled mold (Figure 2). After purging the air and stabilizing the coolant flow, the molten metal is poured into the mold following thorough stirring. After cooling to room temperature, the Zn-22Al alloy ingot is obtained.

The cast ingot undergoes air-cooling and is subsequently placed in a box-type resistance furnace set to 360 °C for 48 h for homogenization annealing. The solubility of zinc in aluminum, ranging from infinity in the liquid state to 2% at room temperature, necessitates this prolonged heat treatment. Rapid cooling during solidification results in an uneven microstructure, thus the extended annealing ensures full diffusion of the α solid solution phase, achieving the most uniform microstructure possible. Following annealing, the resistance furnace is set to 0 °C, and the cast ingots are cooled to room temperature inside the furnace. Finally, the ingots are aged at room temperature for 3 days.

### 2.2. Hot Rolling of the Zn-22Al Alloy Plates

The primary deformation mechanism in Zn-Al alloys is grain boundary sliding, and refining the grain size of Zn-22Al alloy is effective in increasing the area of grain boundaries. This, in turn, facilitated easier sliding and enhanced superplastic deformation capability [27], as indicated by Kurosawa et al.’s research. Two main mechanical processing strategies are commonly employed to achieve superplasticity in Zn-Al alloy ingots: Equal Channel Angular Pressing (ECAP) and Thermomechanical Controlled Processing (TMCP), with the latter involving hot rolling. Among these methods, TMCP is more readily implemented and well-suited for producing large-size dampers for seismic energy dissipation in building structures.

In this study, the ingots obtained in the previous section underwent hot rolling following solid solution treatment (held at 360 °C for 4 h). The elevated temperature promotes atomic diffusion within the alloy, reducing segregation and aiding in the welding of vacancies, resulting in compaction and reduced porosity.

Before rolling, all surface defects were removed from the ingots to achieve a uniform thickness and produce regular metal ingots. Due to the absence of heating equipment in the rolling mill, the alloy plate underwent a re-heating process in a resistance furnace adjacent to the rolling mill at 360 °C for 15 min after each reciprocating rolling. This ensured that the alloy temperature remained within the range of 300~360 °C.

The reduction ratio is a crucial process parameter for achieving superplasticity in Zn-Al alloys, with literature indicating that an increase in the reduction ratio improves the plastic deformation capability of the alloy, as research by Dong et al. [28]. In this study, the alloy ingots were rolled from 11 mm to 3 mm along the thickness direction, resulting in a total reduction rate of 72.7%, with a reduction ratio below 20% in each roll. The diameter of the rolling mill’s roller was 180 mm, rotating at a speed of 12 rpm. No lubricant was applied to the roller during the rolling process, which was conducted in a reciprocating manner. Subsequently, all rolled plates were air-cooled.

### 2.3. Heat Treatment of the Zn-22Al Alloy Plates

After aging at room temperature for 3 days, the final stage of the Zn-22Al alloy preparation involves heat treatment. The first step is the solid solution process, where the plates obtained from the previous step are placed inside a resistance furnace at 360 °C for 6 h. This solid solution process aims to enhance the microstructure uniformity of the alloy and eliminate any detrimental effects of superplasticity resulting from the non-uniformity of the microstructure after hot rolling.

The second step in this phase is quenching. Costa et al.’s research indicated that the supersaturated α’ phase decomposed into fine equiaxed two-phase eutectoid structures at temperatures below 50 °C [29]. Lower temperatures lead to a more uniform structure during the decomposition of the α’ phase, resulting in finer grains, increased equal-axing, and enhanced favorability for the superplasticity of the alloy. In this process, the alloy plates, previously heated to 360 °C inside a resistance furnace, are directly immersed in room temperature water to complete the quenching process. After complete water cooling, the third step involves removing the alloy plates from the water and allowing them to age at room temperature for an additional three days. Following this step, the production of the alloy plates is complete.

## 3. Material Test of the Zn-22Al Alloy

In this section, material-level tests were conducted to investigate the basic material properties and fatigue performance of Zn-22Al alloy. The final number of specimens under cyclic loading is determined based on experimental results, ensuring that at least two sets of usable data are available for each strain amplitude after excluding invalid data. A total of 16 specimens were tested with different loading modes and strain amplitudes, 9 sets of data were presented as the basis for subsequent analysis. Monotonic tensile curves and cyclic stress–strain curves were obtained from valid test results.

### 3.1. Test Arrangements and Design

**Figure 3:**
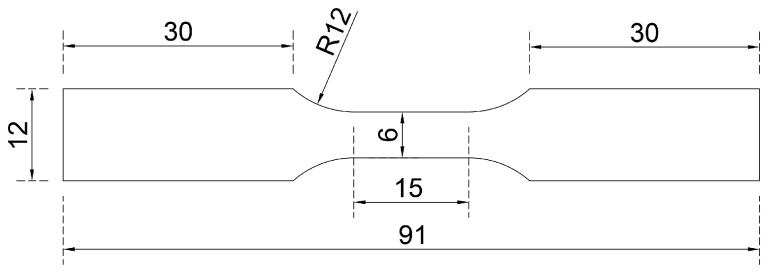
Geometry and dimensions of test specimens.

Monotonic tensile tests and LCF tests of Zn-22Al alloy were carried out to contribute to the limited data currently available on seismic energy dissipation materials used in seismic dampers. As mentioned in the previous section, a plate-shaped energy dissipation metal is more suitable for seismic dampers. Therefore, the specimens utilized in this study were plate-shaped and wire EDM-cut from the plates obtained in Section 2.3, following the standards of ASTM E606/E606M-21 as well as Chinese standards (GB/T 24172-2009, GB/T 228.1-2021), as illustrated in Figure 3.

The specimen geometry was designed to ensure that fracture is anticipated within the central parallel segment. Each specimen received a unique identifier, commencing with the loading type and followed by the strain amplitude. The details of the material test specimens are succinctly presented in Table 1. As research by Hong et al., Ahmad and Ajaj, and Saini et al. indicated, the loading frequency had a remarkable effect on fatigue performance [30,31,32], and the chosen loading frequency adequately covered the strain rate of energy dissipation materials inside seismic damping devices during earthquake actions.

**Figure 4:**
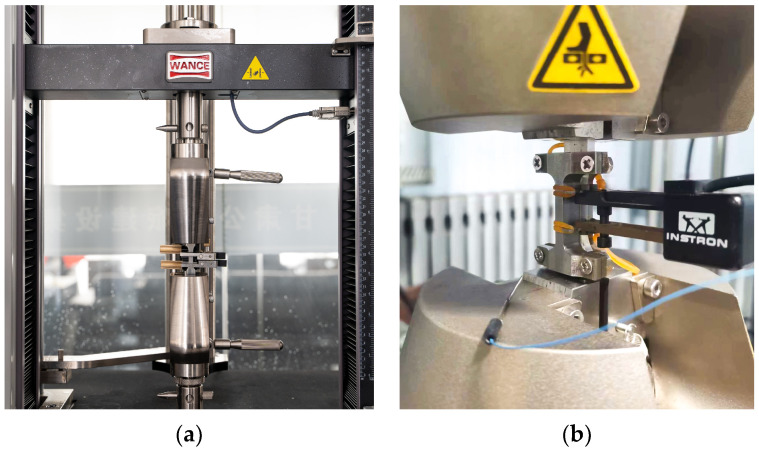
Zn-22Al alloy material tests: (**a**) Test setup for monotonic tests, (**b**) Test setup for fatigue tests.

For monotonic tests, specimens were firmly secured using wedge jaws on a universal material testing machine equipped with a long-stroke extensometer. The tests were executed under strain control, applying a strain rate of 2 × 10^−3^/s along the pull direction until failure, as illustrated in Figure 4a. The loading direction aligns with the rolling direction.

In LCF tests, specimens were clamped using the hydraulic wedge jaws of an INSTRON 8801 servo hydraulic fatigue testing system, with aluminum spacers positioned at both ends of the specimens inside the jaws. The tests were conducted under strain control at a rate of 2 × 10^−3^/s, employing a constant strain amplitude (ranging from ±0.8% to ±2.5%) until the specimen fractured, as depicted in Figure 4b. Dynamic extensometers with a gauge length of 12.5 mm were employed in the fatigue tests.

The Zn-22Al alloy, characterized by a low hardening coefficient under cyclic loading, possesses remarkable energy dissipation capabilities. However, this lower hardening coefficient makes the material susceptible to crack propagation and defects, leading to potential local buckling and partial failure. Using this characteristic, the Zn-22Al alloy can be strategically designed as an energy-dissipation metal within seismic dampers that incorporate buckling constraints.

**Figure 5:**
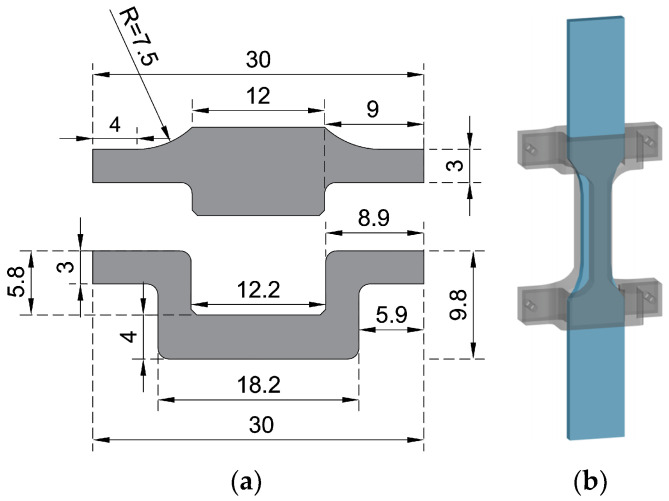
Anti-buckling fixture: (**a**) Top view, (**b**) Side view.

Considering the material’s characteristics and its application scenarios, an anti-buckling fixture, as depicted in Figure 5, was designed for specimens undergoing cyclic tests. The fixture design referenced the Chinese standard GB/T 3075-2021 [33]. Two parts of the fixture were securely fastened together using screws and nuts, with special design details ensuring that the fixture does not impede the specimen’s longitudinal sliding within it. This design prevents out-of-plane buckling of the specimen inside the fixture and ensures that it does not interfere with the clamping of the dynamic extensometer.

### 3.2. Monotonic Tensile Tests

**Figure 6:**
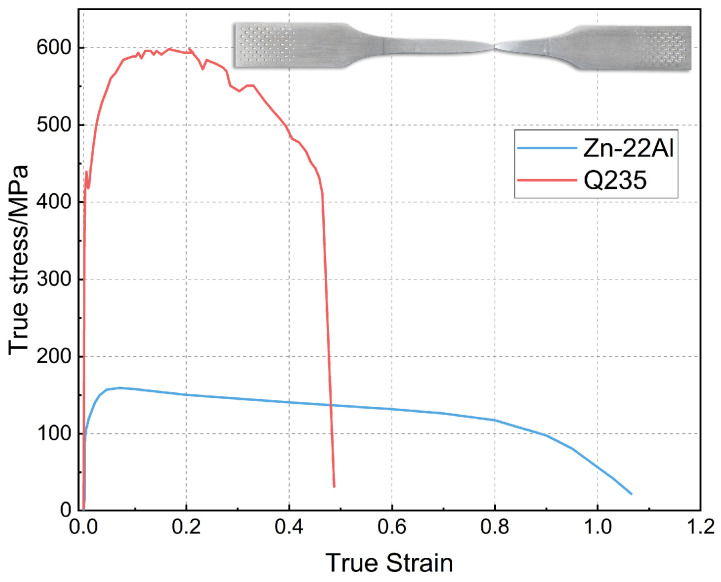
Stress–strain curves under monotonic loading.

The true stress–strain curves of the Zn-22Al alloy, derived from the engineering stress–strain data of the monotonic tensile test, are illustrated in Figure 6. For comparison, a stress–strain curve of mild steel (Q235) was included, obtained at the same strain rate of 2 × 10^−3^/s. The monotonic test results indicate that mild steel typically exhibits a distinct yield plateau before hardening, while the Zn-22Al alloy demonstrates continuous yielding behavior and remarkable plastic deformation capability.

**Figure 7:**
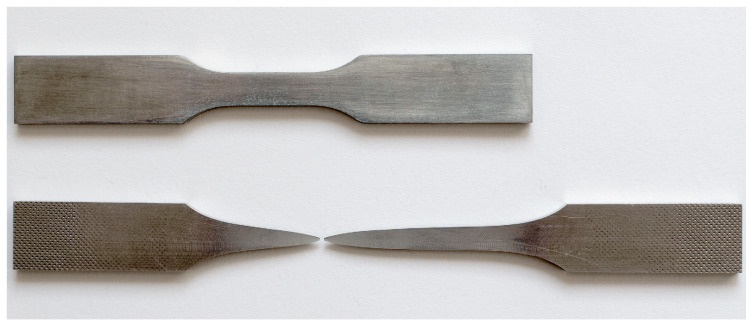
Specimens both before and after the monotonic tensile test.

Due to the continuous yielding behavior of the Zn-22Al alloy, the 0.2% proof strength is utilized as the equivalent yield strength. The equivalent yield strength of the Zn-22Al alloy is only 40 MPa, and it undergoes prolonged steady-state flow after yielding until fracture. The low yield strength allows the Zn-22Al alloy to enter the working state earlier under loading, enabling dampers made from it to cover a wider operational range. The extended steady-state flow provides dampers made from it with a greater reserve of plastic deformation capability under extreme loading conditions. The percentage elongation at fracture ε_f_ is defined as the percentage elongation over the standard gauge length. As shown in Figure 6, ε_f_ reaches 200.37%, reflecting a fracture strain of 1.12. Figure 7 visually represents specimens both before and after the monotonic tensile test.

The Zn-22Al alloy also exhibits less strain hardening and greater ratios of ultimate tensile strength to yield strength, which shows great potential in energy dissipation and a significant reserve of strength. These unique properties make Zn-22Al alloy suitable for seismic dampers, especially in situations with high deformation and high fatigue life demands. It is worth noting that, due to the alloy’s less strain hardening characteristics, additional measures to prevent local buckling are necessary when designing seismic dampers using Zn-22Al alloy. A summary of the basic material properties of Zn-22Al alloy is presented in Table 2. The parentheses following the data in the table indicate the relative error compared to the average value.

The comparison of material properties between Zn-22Al alloy and common energy dissipation metals is presented in Table 3. As common energy dissipation materials, Q235 (mild steel) and BLY160 (low yield point steel) both show high yield strength and low fracture elongation. Comparing the data in Table 3, it is evident that material performance data for superplastic Zn-22Al alloy under conditions of room temperature, high strain rates, and plate-shaped specimens are still lacking.

### 3.3. Low-Cycle Fatigue Tests

In this section, the results of the constant strain amplitude fully reversed cyclic axially loaded tests are presented and analyzed. Cyclic stress–strain curves are provided, and strain-life relationships are calibrated using the Coffin-Manson relationship.

#### 3.3.1. Cyclic Stress–Strain Curves

Traditionally, three methods are used for the construction of cyclic stress–strain (CSS) curves: the multiple-step, incremental-step, and companion methods. While the companion method requires larger sample numbers compared to the former two (as it requires only one sample), it emerges as the method of choice for materials characterized by lower fatigue life. In the present study, the companion method was utilized to construct the CSS curves, with each specimen subjected to loading at a constant strain amplitude.

**Figure 8:**
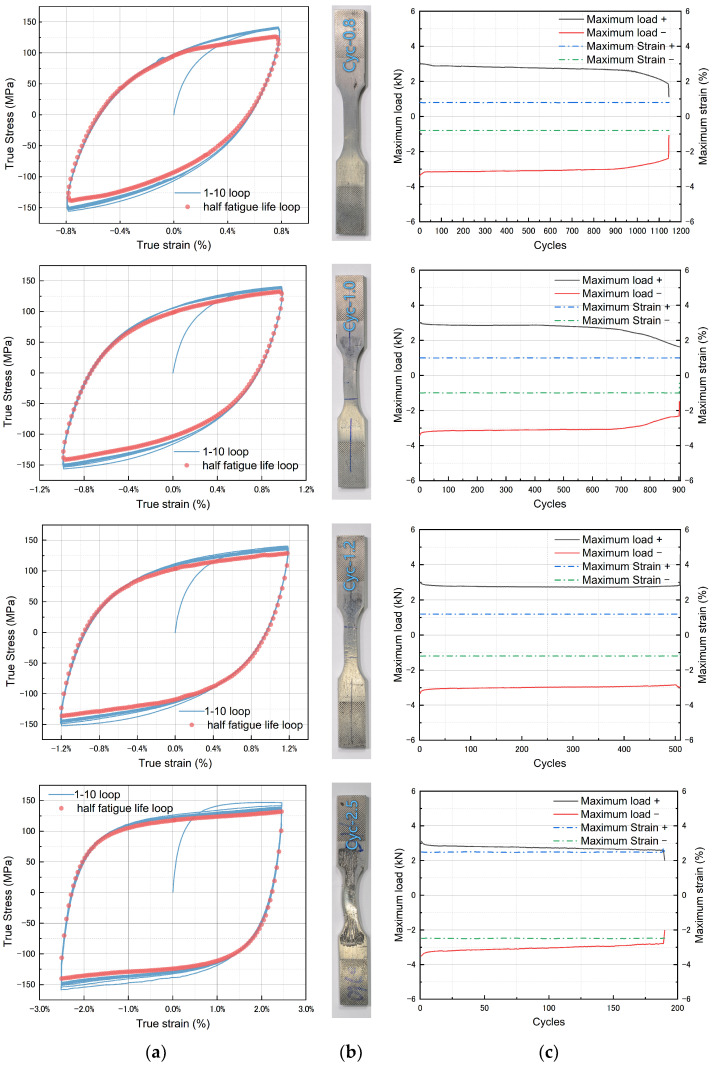
(**a**) Stress–strain curves of test specimens, (**b**) specimens after tests, and (**c**) peak force during fatigue tests.

**Figure 9:**
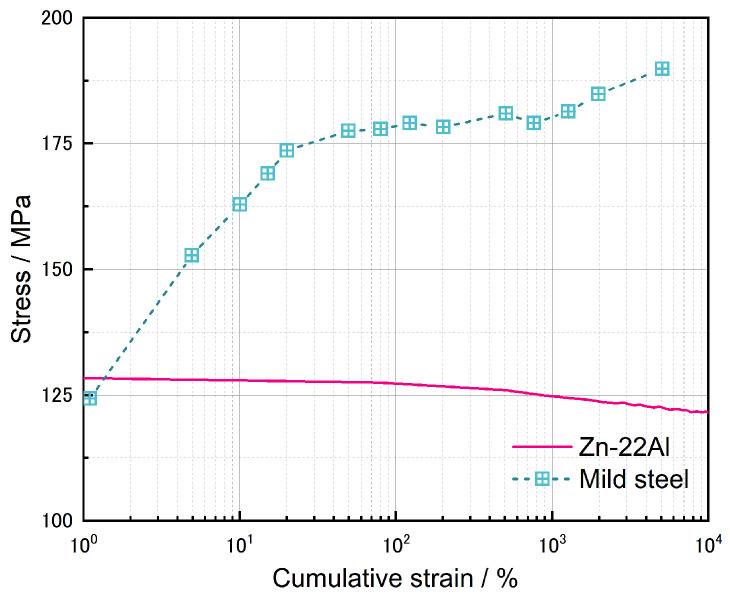
The evolution of stress as a function of cumulative strain for Zn-22Al alloy and mild steel.

Figure 8a illustrates the CSS curves for the first 10 cycles and the cycles corresponding to half of the fatigue life for each specimen. The variation of peak tensile and compressive loads with the number of cycles for each specimen is shown in Figure 8c. From Figure 8a, it can be observed that the hysteresis loops of the Zn-22Al alloy are full and symmetric. Under cyclic loading, the Zn-22Al alloy exhibits slight cyclic softening, and the material response stabilizes within the first few cycles. The rate and degree of cyclic softening can be illustrated by examining the evolution of stress amplitudes at each cycle during the tests. This is depicted for the four considered strain amplitudes of Zn-22Al alloy in Figure 8c. The strain hardening behavior is evident in mild steel under cyclic loading conditions, as shown in Figure 9, while Zn-22Al alloy exhibits a subtle strain softening from another perspective [26].

It should be noted that despite the implementation of anti-buckling fixtures and the carefully designed specimen geometry to prevent specimen buckling, the specimen still experienced in-plane buckling, particularly under cyclic loading with larger strain amplitudes. Fortunately, despite some degree of in-plane buckling occurring, there was no occurrence of out-of-plane buckling, and the specimen displayed a stable hysteresis behavior, as depicted in Figure 8. Therefore, unlike typical fatigue tests, the fatigue tests in this study were set to terminate either when the peak load of the specimen decreased to below 65% of its maximum value or when significant failure occurred in the specimen.

The CSS curve could be described by Ramberg–Osgood models, written in terms of stress and strain amplitudes, as given by

(1) $$\frac{\Delta \varepsilon }{2}=\frac{\Delta \varepsilon_{e}}{2}+\frac{\Delta \varepsilon_{p}}{2}=\frac{\Delta \sigma }{2E}+{\left(\frac{\Delta \sigma }{2K^{\prime }}\right)}^{\frac{1}{n^{\prime }}}$$

where $\frac{\Delta \varepsilon }{2}$ is the total strain amplitude, $\frac{\Delta \varepsilon_{e}}{2}$ is the elastic strain amplitude, $\frac{\Delta \varepsilon_{p}}{2}$ is the plastic strain amplitude, Δσ is the stabilized stress amplitude at half fatigue life cycle, *E* is the Young’s modulus, $K^{\prime }$ is the cyclic strength coefficient, and $n^{\prime }$ is the cyclic strain hardening exponent. The material constants were obtained through a power-law regression curve fitting to stress amplitude versus plastic strain amplitude data, resulting in $K^{\prime }=138.17$ and $n^{\prime }=0.061$.

**Figure 10:**
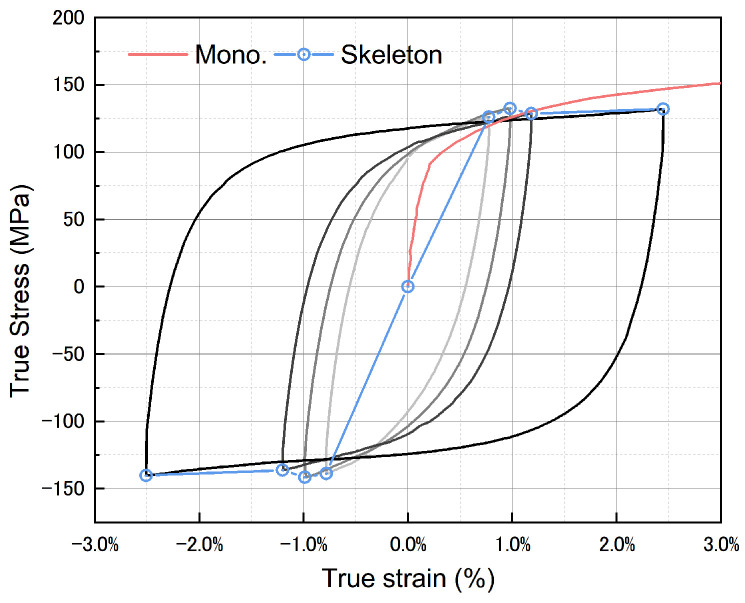
Cyclic skeleton curve and monotonic stress–strain curves.

The cyclic skeleton curve, constructed by connecting the tips of stabilized loops at different strain amplitudes, as shown in Figure 10, reveals that the Zn-22Al alloy exhibits a phenomenon of cyclic hardening followed by cyclic softening. At small strain amplitudes, the cyclic stress is slightly higher than that in the monotonic tensile test. With increasing strain amplitude, the cyclic stress becomes lower than the monotonic tensile test. At the minimum considered strain amplitude of 0.8%, the stress in the cyclic stress–strain curve is approximately 4% higher than the monotonic tensile test value. For the maximum considered strain amplitude of 2.5%, the stress in the cyclic stress–strain curve is approximately 12% lower than the monotonic tensile test value.

**Figure 11:**
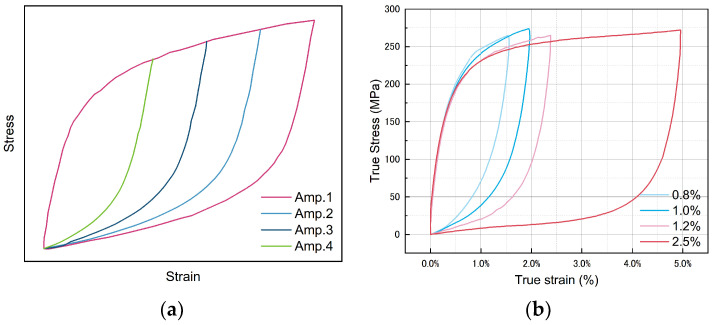
(**a**) Schematic diagram of Masing behavior and (**b**) non-Masing behavior of Zn-22Al alloy.

Aligning the starting points of the ascending branches of stable hysteresis loops with different strain amplitudes to the same position in the same figure allows us to assess the material’s behavior. Masing behavior, characterized by completely overlapping ascending branches of hysteresis loops (Figure 11a), signifies kinematic hardening [40]. However, as shown in Figure 11b, Zn-22Al alloy does not exhibit the characteristics of kinematic hardening. This suggests that the alloy may demonstrate isotropic hardening or mixed hardening characteristics.

#### 3.3.2. Strain–Life Relationship

**Figure 12:**
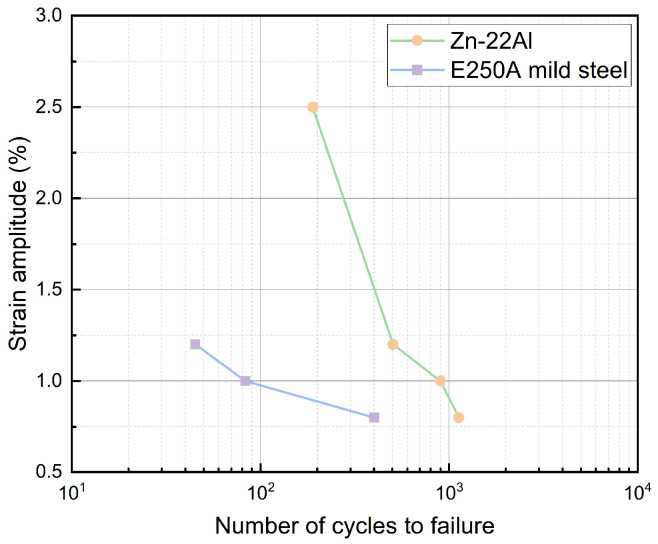
Low-cycle fatigue life of the Zn-22Al alloy and mild steel.

The fatigue life of the Zn-22Al alloy under various strain amplitudes is depicted in Figure 12, with a typical mild steel E250A curve also featured in the figure for comparison [41].

Fatigue tests are commonly recognized to exhibit considerable scatter, even under meticulously controlled conditions. The Zn-22Al alloy used in this study, with its soft structure, introduces some inevitable variability. It is crucial to highlight that the extensometer notch can impact results if improperly fastened. Additionally, the challenges faced by plate specimens under cyclic loads differ significantly from the geometric symmetry of cylindrical specimens. Consequently, there is a notable difference in fatigue life between plate and cylindrical specimens. Zhang et al.’s research emphasized the influential role of specimen geometry in the fatigue performance and crack initiation of alloys [42]. Specifically, specimens with the same geometry but different reduced working section lengths demonstrate a noteworthy influence on fatigue life [24,43].

The statistical data on the fatigue life of common energy dissipation metals in relevant studies, as well as the fatigue life data for Zn-22Al alloy in the present work, are presented in Table 4. It should be noted that a total of 13 specimens were tested, out of which 11 specimens underwent cyclic loading. Data from specimens where failure did not occur in the gripping region of the extensometer and from specimens that experienced unexpected failure due to processing defects were also excluded. Therefore, 6 sets of data were selected (including 2 sets for monotonic tensile tests) and presented as the basis for subsequent analysis. From Table 4, it is clear that the Zn-22Al alloy exhibits superior fatigue life compared to common metals, including mild steel, low-yield point steel, and stainless steel. Despite the multiple adverse factors mentioned above, the fatigue life of the Zn-22Al alloy plates still reaches 2.25 to 6.07 times that of E250A mild steel (cylindrical specimens).

The total strain amplitude can be decomposed into elastic strain amplitude and plastic strain amplitude. It has been observed that plastic strain-life data from LCF tests roughly follow a straight line when plotted on a log-log scale. This observation forms the basis of the basic Coffin-Manson relationship [46], expressed as:

(2) $$\frac{\Delta \varepsilon_{p}}{2}={\varepsilon^{\prime }}_{f}{\left(2N_{f}\right)}^{c}$$

where $\Delta \varepsilon_{p}$ is the plastic strain amplitude, ${\varepsilon^{\prime }}_{f}$ is the fatigue ductility coefficient, *c* is the fatigue ductility exponent and $2N_{f}$ is the number of reversals to failure.

By incorporating the elastic strain amplitude, a Basquin-Coffin-Manson relationship can be established, expressed as:

(3) $$\frac{\Delta \varepsilon }{2}=\frac{\Delta \varepsilon_{e}}{2}+\frac{\Delta \varepsilon_{p}}{2}=\frac{{\sigma^{\prime }}_{f}}{E}{\left(2N_{f}\right)}^{b}+{\varepsilon^{\prime }}_{f}{\left(2N_{f}\right)}^{c}$$

where ${\sigma^{\prime }}_{f}$ and *b* are the fatigue strength coefficient and fatigue strength exponent, respectively.

**Figure 13:**
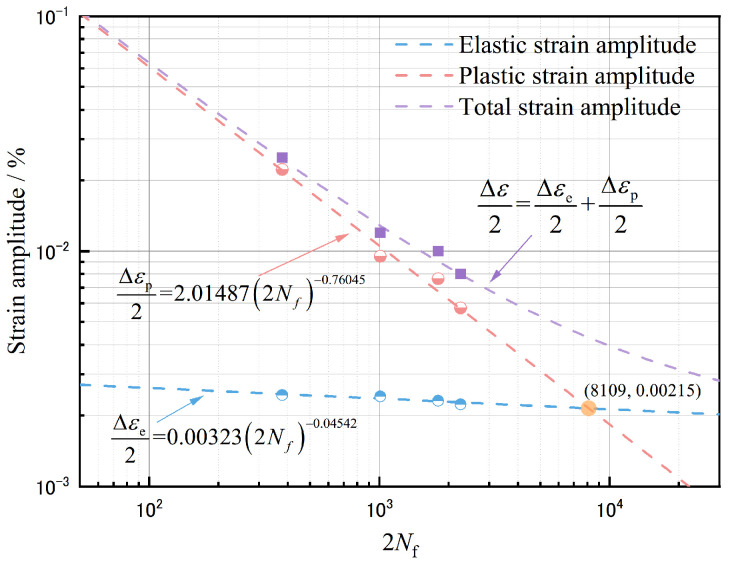
Low-cycle fatigue characteristics of the Zn-22Al alloy.

In Table 4, the fatigue life of the Zn-22Al alloy is listed for four different strain amplitudes. The total strain amplitudes can be divided into elastic and plastic parts, as expressed in Equation (3) of the Basquin-Coffin-Manson relationship. The elastic strain amplitude can be obtained by dividing the stress amplitudes by Young’s modulus E, and the plastic strain amplitude can be obtained by subtracting the elastic strain amplitude from the total strain amplitudes. With four data pairs of ($\Delta \varepsilon_{e}/2$, $2N_{f}$) for the elastic part and ($\Delta \varepsilon_{p}/2$, $2N_{f}$) for the plastic part, the fatigue strength coefficient ${\sigma^{\prime }}_{f}$, fatigue strength exponent *b*, fatigue ductility coefficient ${\varepsilon^{\prime }}_{f}$, and fatigue ductility exponent *c* can be fitted. Based on the fitted parameters, the Basquin-Coffin-Manson relationship is depicted in Figure 13 with trend lines showing the variations of elastic, plastic, and total strain amplitudes with the number of reversals to failure.

Despite some scattering, the results generally suggest that the Basquin-Coffin-Manson relationship is applicable to the Zn-22Al alloy. From Figure 13, it can also be observed that the elastic strain constitutes a relatively small proportion of the total strain amplitude. Therefore, fatigue life is primarily influenced by the plastic strain amplitude.

The transition fatigue life point, determined by the intersection between the fitted elastic strain and plastic strain lines, is approximately at 8109, 0.00215. This once again confirms that the strain amplitude considered in this study leads to fatigue behavior dominated by LCF or ELCF, in contrast to high cycle fatigue (HCF). In HCF tests, the elastic strain amplitude is higher than the plastic strain amplitude, and the number of cycles to failure generally ranges up to 10^6^.

**Figure 14:**
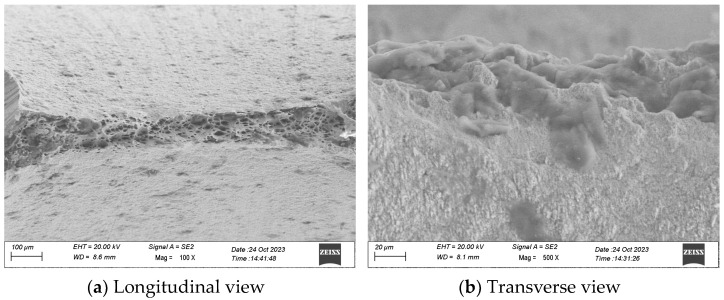
SEM images of the fracture surface of a monotonic tensile specimen.

Different test categories correspond to different failure modes, wherein higher strain amplitudes predominantly result in a ductile failure mechanism, while lower strain amplitudes primarily manifest a fatigue failure mechanism. The ductile damage failure mechanism typically prevails when the number of cycles to failure is below 100–200. Failure induced by ductile damage yields a relatively uneven fracture surface, often displaying a distinctive cup-and-cone profile, as exemplified in the scanning electron microscope (SEM) figure of the monotonic tensile specimen in Figure 14.

On the other hand, fatigue failure is characterized by a sequence of beach marks, serving as indicators of the progressive advancement of a fatigue crack. Specimens that failed within the Low-Cycle Fatigue (LCF) regime exhibited either ductile damage or a mixed-mode failure. This shift in the damage mode might contribute to the variability observed in the test results [45].

### 3.4. Energy Dissipation

The energy dissipation capability of the specimen can be defined by the dimensionless index—equivalent viscous damping (EVD) ratio, as shown in the following equation:

(4) $$\zeta_{\mathrm{eq}}=\frac{E_{D}}{4\pi E_{E}}$$

where $E_{D}$ is the area of the steady hysteretic loop which is mentioned in Figure 8, and $E_{E}$ is the maximum elastic strain energy. The EVD ratios, calculated for various strain amplitudes, fall within the range of 0.372 to 0.512. The EVD for Zn-22Al alloy is slightly lower than that for common metals, as the tests for mild steel exhibited more strain hardening, resulting in a larger $E_{E}$. Conversely, the absolute energy dissipated by Zn-22Al alloy per cycle is greater than that for mild steel.

## 4. Microstructures of the Zn-22Al Alloy

**Figure 15:**
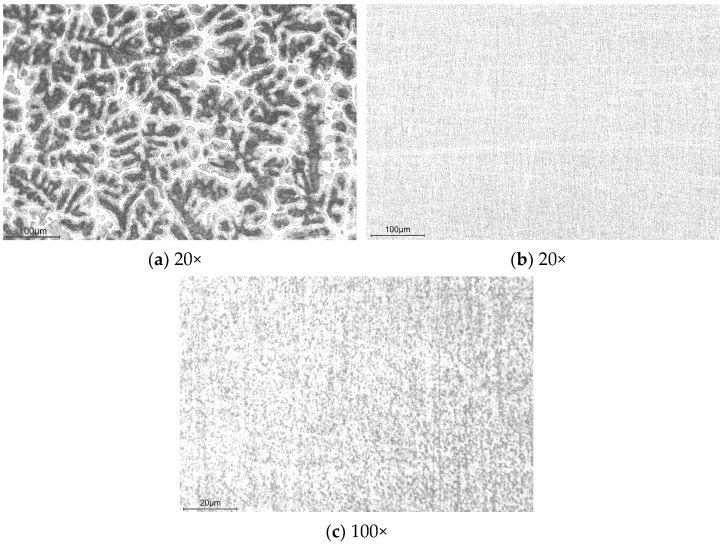
Optical micrographs of (**a**) As-cast and (**b**,**c**) Treated samples in different magnifications.

The microstructure of the casted ingot obtained in Section 2.1 was analyzed using an optical microscope. Samples for microstructural analysis underwent grinding with various roughness SiC papers and polishing with W2.5 diamond paste. Subsequently, they were etched with a 1 vol.% hydrofluoric acid solution. Optical micrographs of Zn-22Al alloy samples, with and without thermomechanical processing, are presented in Figure 15. The aluminum-rich areas, being more susceptible to hydrofluoric acid corrosion than zinc, appear black, while the zinc-rich areas appear bright. As depicted in Figure 15a, the as-cast sample displays a coarse dendritic microstructure.

Following the various processes detailed in the previous section, the coarse dendritic microstructure disappears in treated samples. As shown in Figure 15b,c, the microstructure of the alloy after processing has been refined to the point where it is indistinguishable under optical microscopy. Studies by Mishra et al., Cetin et al., and Demirtas et al. demonstrated that the Zn-22Al alloy’s maximum elongation and optimal strain rate increased with decreasing grain size [47,48,49].

**Figure 16:**
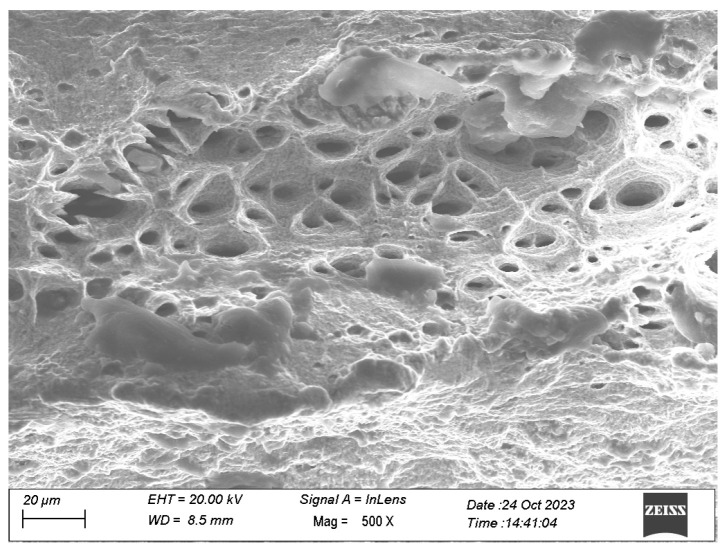
SEM figure of the fracture surface of a monotonic tensile specimen.

As illustrated in Figure 16, the fracture surface confirms the ductile fracture characteristics exhibited by the specimen under monotonic tensile loading. The prevalent dimpled pattern observed across the entire fracture surface signifies a microvoid growth and coalescence fracture mechanism.

## 5. Parameter Calibration and Finite Element Simulation

Equation (1) generally reflects the material’s response to stable cycles under cyclic loading at different strain amplitudes. However, for a comprehensive numerical simulation study, an appropriate constitutive material model capable of accurately predicting the material response under cyclic loading is required.

Based on a comprehensive study by Lemaitre and Chaboche [50], the combined cyclic hardening material model in the nonlinear finite element software ABAQUS was employed in this study. This model combines the isotropic and kinematic hardening effects rationally, enabling uniform expansion and translation of the yield surface in the stress space simultaneously.

**Figure 17:**
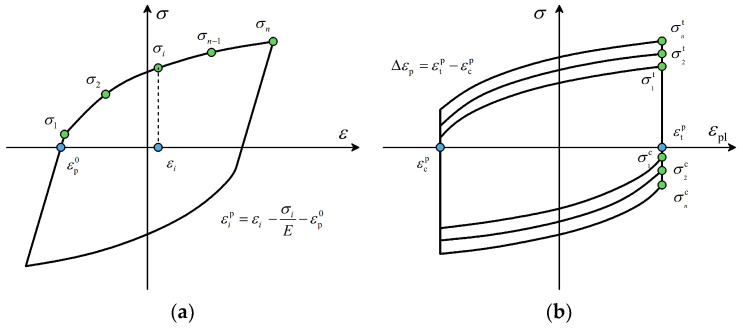
Calibration of the combined cyclic hardening material model parameters: (**a**) Kinematic component, (**b**) Isotropic component.

In this model, the isotropic component defines the change in the size of the yield surface σ as a function of equivalent plastic strain $\varepsilon^{p}$ and is given by:

(5) $$\sigma =\sigma {|}_{0}+Q_{\infty }\left(1-e^{-b_{\mathrm{iso}}\varepsilon^{p}}\right)$$

where $\sigma {|}_{0}$ is the yield stress at zero equivalent plastic strain (defined in this study as the 0.2% proof stress), $Q_{\infty }$ is the maximum change in the size of the yield surface, and $b_{\mathrm{iso}}$ is the rate at which the size of the yield surface changes as plastic strain increases. The size of the yield surface in the *i*^th^ cycle $\sigma_{i}$ can be obtained from:

(6) $$\sigma_{i}=\frac{\sigma_{i}^{t}-\sigma_{i}^{c}}{2}$$

where $\sigma_{i}^{t}$ and $\sigma_{i}^{c}$ are the maximum tensile and compressive stresses of the *i*^th^ cycle, as shown in Figure 17.

The equivalent plastic strain corresponding to $\sigma_{i}$ is:

(7) $$\varepsilon_{i}^{p}=\frac{\Delta \varepsilon_{p}}{2}\left(4i-3\right)$$

in which $\Delta \varepsilon_{p}$ is the plastic strain range as illustrated in Figure 17b.

At this point, Equation (5) can be fitted through each data pair $\left(\sigma_{i},\varepsilon_{i}^{p}\right)$.

The kinematic hardening component of the model defines the change of backstress α, which is expressed as:

(8) $$\alpha =\sum_{k=1}^{n}\frac{C_{k}}{\gamma_{k}}\left(1-e^{-\gamma_{k}\varepsilon^{p}}\right)$$

in which *C* and *γ* are the constants that need calibration. Specifically, the *C/γ* ratio determines the maximum change in the back stress and *γ* describes the rate at which the backstress changes with plastic strain increase. Data pairs $\left(\alpha_{i},\varepsilon_{i}^{p}\right)$ are obtained considering the following coordinate translation rule:

(9) $$\varepsilon_{i}^{p}=\varepsilon_{i}-\frac{\sigma_{i}}{E}-\varepsilon_{p}^{0}$$

where $\varepsilon_{p}^{0}$ is the value of the smallest plastic strain at zero stress. For each data pair, the corresponding backstress is obtained from:

(10) $$\alpha_{i}=\sigma_{i}-\frac{\sigma_{1}+\sigma_{n}}{2}$$

where $\sigma_{1}$ and $\sigma_{n}$ are the stresses in the first and last data pairs, respectively. At this point, Equation (8) can then be fitted to the pairs of data points $\left(\alpha_{i},\varepsilon_{i}^{p}\right)$. To ensure the accuracy and representativeness of the stress–strain hysteresis curve, the total least squares regression method was employed in this research. This method minimizes errors on both axes, contributing to a more precise depiction of the curve [45].

The material model parameters calibrated for each test specimen are summarized in Table 5. As recommended by the ABAQUS manual [51], two sets of kinematic hardening components have been superposed, which may effectively improve the simulation results. Certain model parameters exhibit variations across different strain amplitudes. This is a prevalent occurrence in metals [52], where the changing hysteretic shapes necessitate distinct combinations of isotropic and kinematic components, each with its associated parameters. In practical applications, engineers can choose a suitable set of calibrated parameters by evaluating the expected working strain range of the material or consider average values directly.

To validate the effectiveness of the combined cyclic hardening material model, finite element simulations were conducted. A plate-shaped model with the same geometry and dimensions as depicted in Figure 3 was employed, utilizing the general-purpose finite element software ABAQUS. Parameters from Table 2 and Table 5 were integrated into the property module as input for both elastic and plastic subpages. The experimentally determined Poisson’s ratio was found to be 0.1667. The three-dimensional eight-node general-purpose reduced integrated (C3D8R) element was adopted in the meshing module. Mesh refinement continued until no significant improvement in simulation results was observed. The simulated model comprised 16,014 elements, with 80 elements set in the transverse axis to accurately capture material behavior.

**Figure 18:**
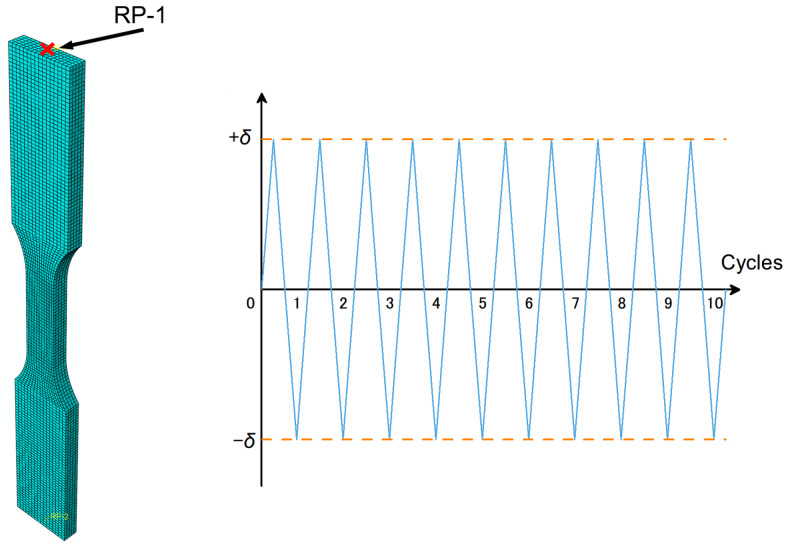
Finite element meshing and cyclic loading pattern of the simulated model.

Cyclic loading, as illustrated in Figure 18, is implemented through the load module using a tabular data under the ‘amplitude’ tab on reference point RP-1. The displacement amplitude (δ) for each load condition is calculated by multiplying the strain amplitude by the parallel length (15 mm). Time intervals are determined by dividing the displacement amplitude by the strain rate. With 10 cycles of cyclic loading applied to the model, the step time for analysis is set as 10 times the cycle time. All degrees of freedom for nodes at both ends are restrained, except for axial displacement at the loading end. To closely emulate real testing conditions, rigid body constraints are enforced between the reference point and the clamping section at both ends of the specimen.

After completing the finite element analyses, stress and strain responses are derived by dividing the reaction force response at the reference point by the cross-sectional area of the specimen (3 × 6 mm^2^) and by dividing the reaction displacement by the parallel length, respectively. These responses facilitate the plotting of the stress–strain (σ − ε) behavior, represented in the form of hysteresis loops.

**Figure 19:**
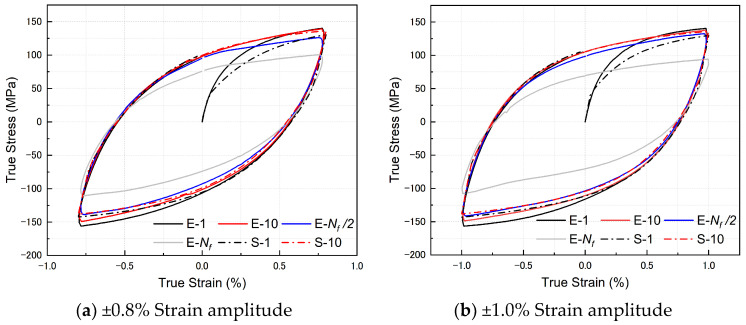

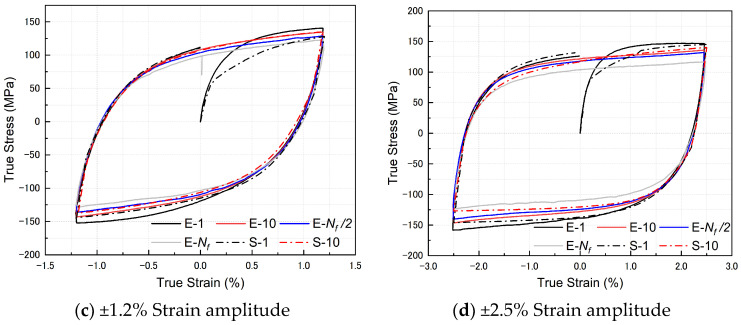
Experimental and FE simulated stress–strain hysteresis curves of Zn-22Al alloy specimens in the first 10 cycles at different strain amplitudes.

The hysteresis loops, predicted by ABAQUS and obtained from experiments, are depicted in Figure 19. In the legend of Figure 19, each hysteresis loop is distinguished by data type and strain amplitudes, where ‘E’ represents experiment and ‘S’ represents simulation. Notably, the finite element method demonstrates remarkable predictive accuracy, closely aligning with experimental values, albeit with a slight deviation in the first loop, which falls marginally below the experimental data. Additionally, a subtle asymmetry is discernible in the hysteresis loops of the four specimens at each strain amplitude. This asymmetry manifests in the lower-left corner of the hysteresis loop, leading to discernible disparities between the finite element simulation results and experimental values. It is plausible that material fatigue or damage may contribute to the observed asymmetry in hysteresis behaviors under cyclic loading conditions.

## 6. Conclusions

The Zn-22Al alloy synthesized in the laboratory underwent comprehensive material testing and analysis, revealing significant insights into its properties and potential applications. The key findings are summarized below:

- Due to the unavailability of commercially produced Zn-22Al alloy, the alloy used in this study was lab-synthesized. The fracture surface of the as-cast sample displayed brittle failure characteristics with extremely low elongation. In contrast, the fully treated alloy exhibited significantly improved elongation, with the fracture morphology indicating a distinct trend of plastic deformation. SEM micrographs supported these findings by showcasing grain refinement, reinforcing the superplasticity of the treated Zn-22Al alloy.
- Monotonic tests revealed that the Zn-22Al alloy demonstrated continuous yielding behavior and exceptional plastic deformation capability. The alloy’s percentage elongation at fracture reached an impressive 200.37%, corresponding to a fracture strain of 1.12. With an equivalent yield strength of only 40 MPa, the alloy displayed prolonged steady-state flow after yielding until fracture. These properties allow dampers made from Zn-22Al alloy to enter the working state earlier and possess a considerable reserve of plastic deformation capability.
- The Zn-22Al alloy exhibited full and symmetrical hysteresis loops, showcasing subtle strain softening under cyclic loading, with stabilization occurring within the initial cycles. Despite challenges posed by its plate-shaped geometry, the alloy displayed an excellent fatigue life—2.25 to 6.07 times that of E250A mild steel (cylindrical specimens). The Ramberg-Osgood model parameters were derived to describe the cyclic stress–strain curve, and the cyclic strain-life relationships aligned well with the conventional Basquin-Coffin-Manson relationship.
- The alloy demonstrated both kinematic and isotropic hardening characteristics under cyclic loading. The parameters of the combined cyclic hardening material model were calibrated, and two sets of kinematic hardening components were superposed to improve simulation accuracy. These parameters were validated to accurately predict the hysteretic behavior of the material using ABAQUS. In practical applications, engineers can select suitable calibrated parameters based on the expected working strain range or consider average values directly.

## Figures and Tables

**Table 1:** Parameters for material test specimens.

Identifier	Loading Type	Frequency	Strain Amplitude	Strain Rate
Mono-1	Monotonic	—	Until broken	2 × 10^−3^/s
Mono-2	Monotonic	—	Until broken	
Mono-3	Monotonic	—	Until broken	
Mono-4	Monotonic	—	Until broken	
Mono-5	Monotonic	—	Until broken	
Cyc-0.8	Cyclic	0.0625 Hz	±0.8%	
Cyc-1.0	Cyclic	0.05 Hz	±1.0%	
Cyc-1.2	Cyclic	0.042 Hz	±1.2%	
Cyc-2.5	Cyclic	0.02 Hz	±2.5%	

**Table 2:** Basic material properties of Zn-22Al alloy.

	Young’s Modulus *E* (GPa)	0.2% Proof Stress (MPa)	Tensile Strength (MPa)	Yield-to-Tensile Ratio	Fracture Elongation (%)
Mono-1	62	43.36 (−0.23%)	164.28 (+1.74%)	3.79 (+1.95%)	215.61 (+7.61%)
Mono-2		41.72 (−4.00%)	154.74 (−4.17%)	3.71 (−0.19%)	187.79 (−6.28%)
Mono-3		42.83 (−1.45%)	159.67 (−1.12%)	3.73 (+0.32%)	213.27 (+6.44%)
Mono-4		44.57 (+2.56%)	163.46 (+1.23%)	3.67 (−1.31%)	195.54 (−2.41%)
Mono-5		44.81 (+3.11%)	165.23 (+2.32%)	3.69 (−0.77%)	189.63 (−5.36%)
Average		43.46	161.48	3.72	200.37

**Table 3:** Superplastic properties of Zn-22Al alloy and common steel.

Material				Superplastic Properties				Ref.
Type	Process	Grain Size (μm)	Shape	T (K)	Yield/Flow Stress (MPa)	Max Elongation (%)	Strain Rate (s^−1^)	
Q235	—	—	Plate	RT	YS:240	35	4 × 10^−3^	[34]
BLY160	—	—	Plate	RT	YS:126	56	—	[8]
Zn-22Al	FSP	1	Plate	RT	FS:180	160	1 × 10^−2^	[35]
Zn-22Al	ECAP	0.3	Cylindrical	RT	FS:150	180	1 × 10^−2^	[36]
Zn-22Al	ECAP	0.35	Cylindrical	RT	FS:90	240	1 × 10^−2^	[37]
Zn-22Al	ECAP	0.25	Plate	RT	—	110	1 × 10^−3^	[38]
Zn-22Al	ECAP	1	Plate	RT	—	195	1 × 10^−3^	[38]
Zn-22Al	TMCP	2.5	Plate	503	10.5	1557	1 × 10^−2^	[39]
Zn-22Al	TMCP	<0.1	Plate	RT	YS:200	180	1 × 10^−2^	[26]
Zn-22Al	TMCP	1.3	Cylindrical	RT	60	~200	1 × 10^−5^	[39]
Zn-22Al	TMCP	—	Plate	RT	43.46	200.37	2 × 10^−3^	Present work

FSP: friction stir processing, ECAP: Equal Channel Angular Pressing, TMCP: Thermomechanical controlling process. YS: Yield stress, FS: Flow stress.

**Table 4:** Summary of cycles to failure for different metal.

Material	Shape	Strain Amplitude					Ref.
		±0.8	±1.0	±1.2	±2.5	±3.0	
Q235 (mild steel)	Cylindrical	—	578	—	—	122	[24]
LY100	Cylindrical	—	512~694	—	123~126	82~119	[44]
LY160	Cylindrical	—	1008	—	139~158	121	[44]
E250A (mild steel)	Cylindrical	400	83	45	—	—	[41]
Stainless steel	Plate	—	671	—	—	39	[45]
Zn-22Al (present work)	Plate	1126	899	504	189	—	—

**Table 5:** Parameters of nonlinear combined isotropic/kinematic hardening model of cyclic plasticity.

Δ*ε*/2 (%)	*σ*|_0_ (MPa)	*Q*_∞_ (MPa)	*b*_iso_	*C*_1_ (MPa)	*γ*_1_	*C*_2_ (MPa)	*γ*_2_
0.8%	39.1	−6.64	3.0	9000	150	31,187	538
1.0%	41.3	−7.81	3.5	6480	108	31,187	538
1.2%	45.0	−9.28	3.5	4680	78	31,187	538
2.5%	82.0	−5.92	3.0	450	3	12,475	215
Mean	51.85	−7.412	3.25	5152.5	84.75	26,509	457.25
COV	0.338	−0.172	0.077	0.605	0.633	0.306	0.306

## Data Availability

The data presented in this study are available on request from the corresponding author.
